# Prevalence and clinical correlates of ascending aortic dilatation in patients with noncompaction cardiomyopathy

**DOI:** 10.1007/s10554-023-02882-2

**Published:** 2023-05-31

**Authors:** Martijn Tukker, Maarten J.G. Leening, Sharida Mohamedhoesein, Alexander L.A. Vanmaele, Kadir Caliskan

**Affiliations:** 1 Department of Cardiology, Erasmus MC University Medical Center Rotterdam, Dr. Molewaterplein 40., Rotterdam, 3015 GD The Netherlands; 2 Department of Epidemiology, Erasmus MC University Medical Center Rotterdam, Rotterdam, The Netherlands

**Keywords:** Noncompaction cardiomyopathy, Dilated cardiomyopathy, Aortic dilatation, Prevalence, Cross-sectional study

## Abstract

Ascending aortic (AoAsc) dilatation can lead to acute aortic syndromes and has been described in various familial cardiac diseases. Its prevalence and clinical significance in patients with noncompaction cardiomyopathy (NCCM) are however unknown. Establishing the prevalence can facilitate recommendations on routine screening in NCCM. In this cross-sectional cohort study based on the Rijnmond Heart Failure/Cardiomyopathy Registry, the patient were enrolment between 2014 and 2021. All NCCM patients (n = 109) were age and sex matched with 109 dilated cardiomyopathy (DCM) patients as controls. The aortic diameters were measured through the parasternal long-axis transthoracic echocardiographic view at the sinuses of valsalva (SoV-Ao), sinotubular junction (STJ) and ascending aorta (AscAo). Dilatation was defined using published criteria adjusted for body surface area (BSA), sex, and age. Median age of age-sex matched NCCM and DCM patients was 45[31–56] vs. 45 [31–55] years with 53% males in both groups. NCCM patients had more familial hereditary patterns and genetic variants (55% vs. 24%, p < 0.001). DCM patients had more heart failure and left ventricular dysfunction (ejection fraction 34 ± 11 vs. 41 ± 12, p = 0.001). Ascending aortic dilatation was present in 8(7%) patients with NCCM and 5(5%) patients with DCM (p = 0.46). All dilatations were classified as mild. In conclusion, in this cross-sectional cohort study the prevalence of ascending aortic dilatation in NCCM patients was 7%, which were only mild dilatations and not significantly different from an age-sex matched cohort of DCM patients. Routine aortic dilatation screening therefore does not seem warranted in patients with NCCM.

## Introduction

Noncompaction cardiomyopathy (NCCM) is characterized by hypertrabeculation of the ventricular walls, and thinning of the compact myocardial layer, resulting in a higher risk of developing heart failure, thromboembolic events, and sudden cardiac death [1]. Consequently, prevention of adverse cardiac events plays a big part in the management of NCCM and extending the life expectancy.

Patients with aortic dilatation are at risk of acute aortic syndromes [2–4]. Research on aortic dilatation in patients with cardiomyopathies is limited. In hypertrophic cardiomyopathies the prevalence of aortic dilatations varies between 4.5 and 18% [5–7]. A study including patients with dilated cardiomyopathy (DCM) compared ascending aortic diameters with a healthy control group (26.6 ± 4.4 vs. 30.6 ± 2.7 mm) [8]. In regard to NCCM, a case series found an association between aortic dilatation in NCCM and the pathogenic variant p.Gly482Arg on HCN4 gene [9]. However, no cohort study has been done on ascending aortic dilatation in NCCM patients to date. Thus, it is necessary to establish if NCCM patients indeed have a higher prevalence of aortic dilatation so routine screening or other precautionary actions against aortic dilatation can be taken.

This study aims to elucidate the prevalence of ascending aortic dilatation in NCCM patients. The secondary objective is to assess if there are associations between the NCCM patients with ascending aortic dilatation, clinical significance, and specific patient characteristics.

## Methods

### Patient selection

This study consisted of 109 NCCM patients previously enrolled in the Rijnmond Heart Failure/ Cardiomyopathy Registry (RHF) included from the Erasmus Medical Centre between February 2014 and January 2021. NCCM patients were matched 1:1 with DCM patients as controls. Matching was based on sex and age within a 5-year range.

Patients were eligible for inclusion if diagnosed with NCCM or DCM, based on clinical presentation, morphologic criteria, and diagnostic imaging in accordance with the current guidelines, and if transthoracic echocardiogram images were available [10–13]. In summary, NCCM was diagnosed in patients with left ventricular wall hypertrabeculation, with a noncompact to compact ratio in parasternal short-axis view in the end-systole was > 2.0 and DCM when dilatation and impaired contraction of the left ventricle or both ventricles are present that are not explained by abnormal loading conditions or coronary artery disease.

### Echocardiographic evaluation

**Fig. 1:**
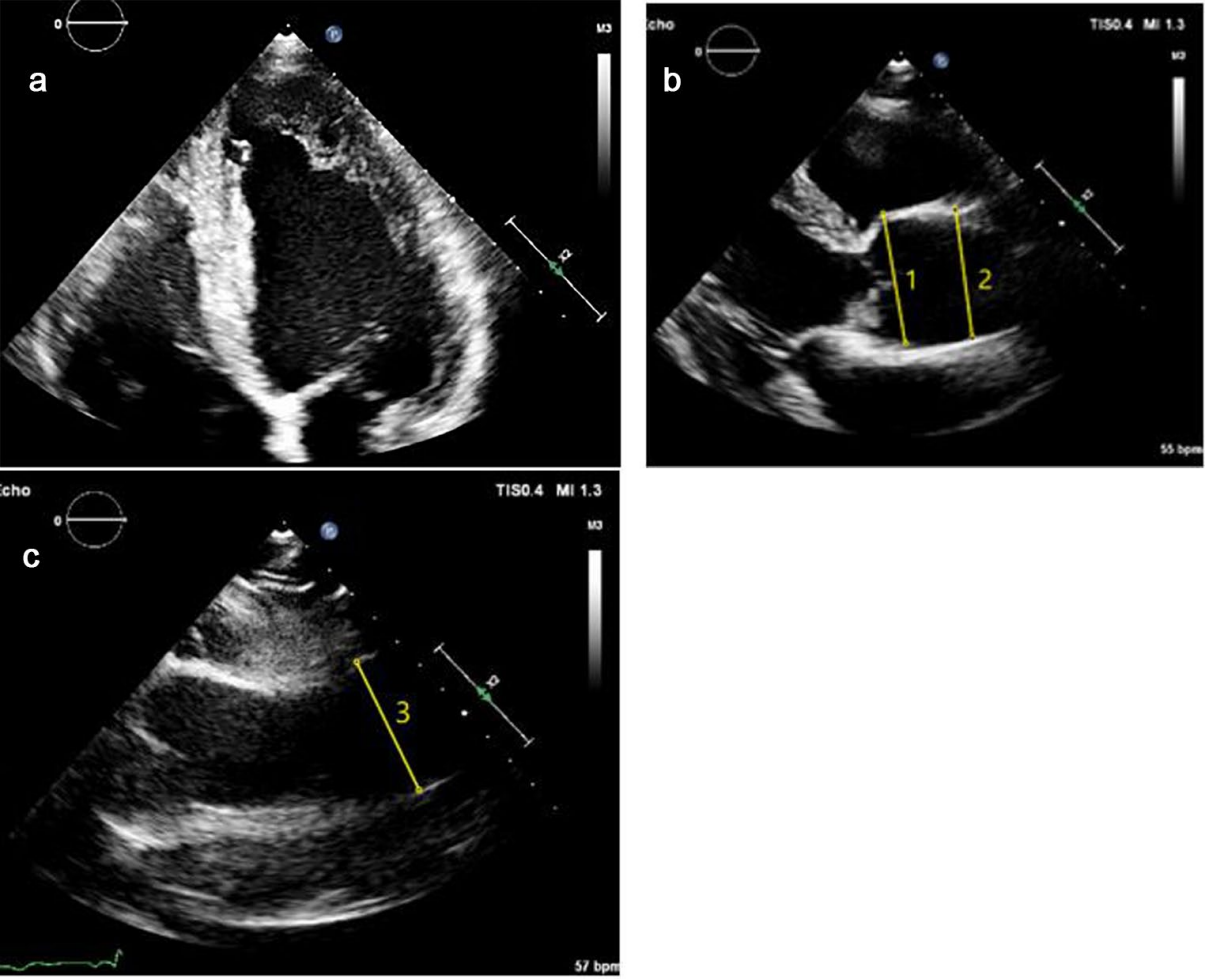
**a, b** and **c**: Measuring technique of the aortic dimensions. Transthoracic echocardiogram images in parasternal long axis view of a 54-year-old male patient with noncompaction cardiomyopathy (NCCM). He had non-sustained ventricular tachycardia at presentation. Figure **1a** shows the hypertrabeculation of the left ventricular myocardial walls. Figure **1b** presents the measurement of the sinuses of valsalva (48 mm) (1), as well as the sinotubular junction (42 mm) (2). Figure **1c** presents the measurement of the ascending aortic diameter, which was 43 mm. The patient had no known familial NCCM and there were no genetic variants found

The first available echocardiography exam after the patient was included in the registry was accessed to measure the following aortic diameters; the sinuses of valsalva (SoV-Ao), the sinotubular junction (STJ) and the maximal diameter of the ascending aorta. Measurements were performed according to the most recent American Society of Echocardiography guidelines from a parasternal long-axis view [14]. The measurements were made at end-diastole, in a strictly perpendicular plane to that of the long axis of the aorta using the leading edge to leading edge convention (Fig. 1).

The quality of the echocardiographic images was scored using the Emergency Ultrasound Standard Reporting Guidelines [15]. A score 2 or lower indicated poor quality.

A dilatation was defined by calculating the personal upper limit of normal, adjusting for sex, age, and body surface area (BSA). The aortic root upper limit of normal was calculated with the formula created by Devereux et al. [16]. The ascending aorta upper limit of normal was adjusted for the same variables age, sex, and BSA according to Ayoub et al. [17].

### Statistical analysis

Normality of distribution was accessed with the Shapiro-Wilk test. Normally distributed continuous data were expressed with mean ± standard deviation, while non-normally distributed data are presented with a median and an interquartile range, and compared with a student’s t-test or Wilcoxon-Mann-Whitney test respectively. Categorical data was expressed with the number of patients and corresponding proportion and compared with a chi-square test or fisher exact test. Univariate and multivariate linear regression analyses were performed to assess the relationship between NCCM and the ascending aortic diameter and presence of ascending aortic dilatation, while adjusting for potential confounders. Multiple imputation was performed to solve missing predictor data prior to the regression analysis. To determine if the data was missing at random, this study used the Little’s MCAR test. Statistical significance is set as two-tailed p-values of < 0.05. Analyses were performed in SPSS (IBM SPSS Statistics, Version 27. Armonk, NY).

## Results

### Baseline characteristics

**Fig. 2:**
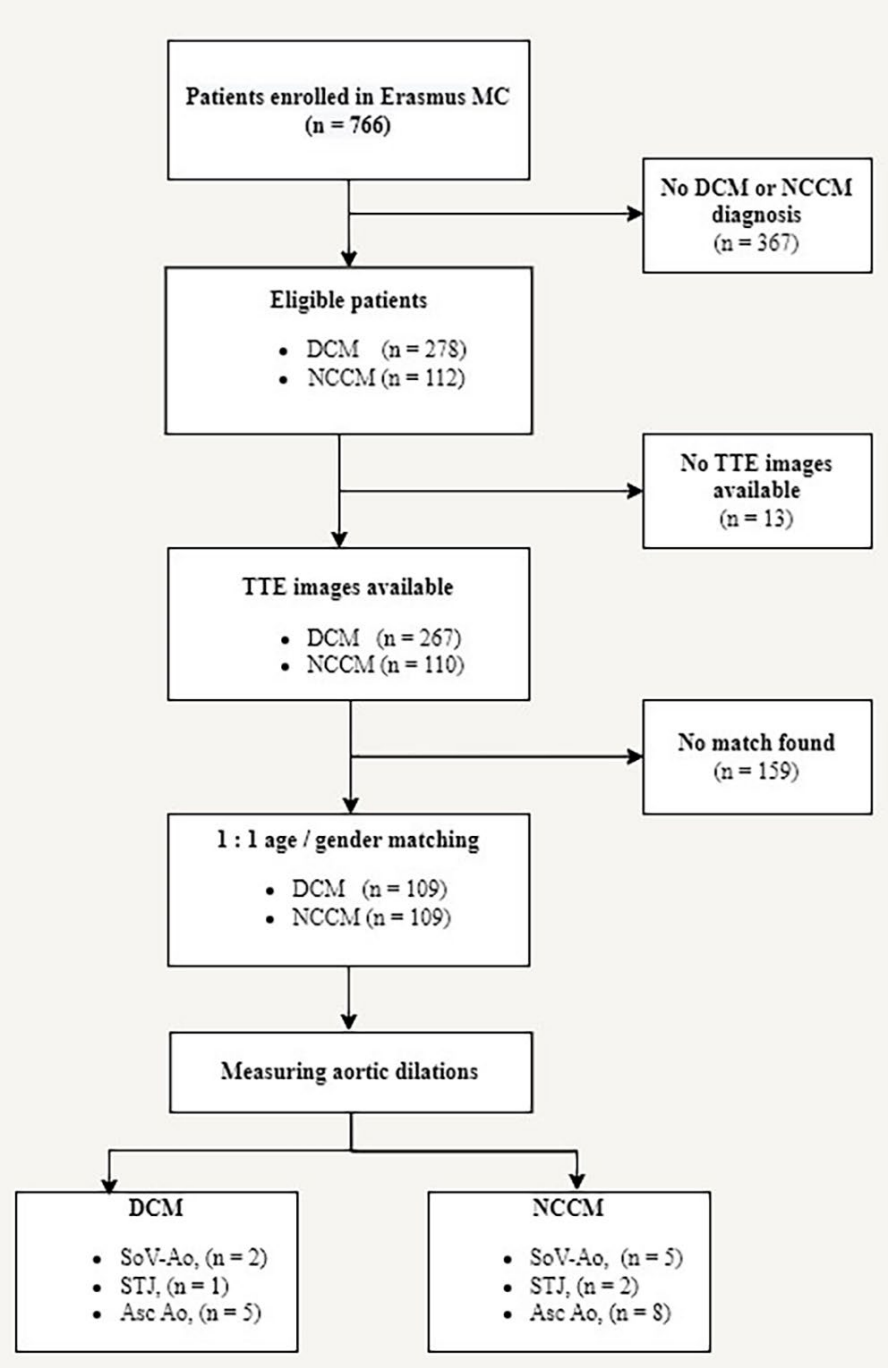
Flowchart study design. RHF = Rijnmond Heart Failure/ Cardiomyopathy Registry; NCCM = Noncompaction cardiomyopathy; DCM = Dilated cardiomyopathy; TTE = transthoracic echocardiography; SoV-Ao = sinuses of valsalva; STJ = sinotubular junction; Asc Ao = ascending aorta; MC = Medical Center

Out of the 766 Erasmus MC patients enrolled in the RHF, 112 were diagnosed with NCCM and 278 with DCM. 110 NCCM patients and 267 DCM patients had available transthoracic echocardiographic images, of which 109 NCCM patients could be matched with 109 DCM patients. Figure 2 Shows a flowchart of the study with the patient selection process.

Characteristics of both patient groups are shown in Table 1. Median age of age-matched NCCM and DCM patients was 45[31–56] vs. 45 [31–55] years (p = 0.94) with 53% males in both groups. This cohort with DCM and NCCM patients had 0 athletes, and included 4 (4%) woman with peripartum cardiomyopathy who all had DCM. Causes of DCM were primarily genetic variants, familial DCM or idiopathic DCM (80%). Nineteen (24%) patients had a positive familial screening for cardiomyopathies. Secondary causes of DCM in this cohort were 2 (2%) patients diagnosed with DCM caused by ethanol or anabolic-androgenic-steroids, 3 (3%) patients with DCM secondary to previous myocarditis, 3 (3%) patients with hypertensive DCM, 9 (8%) patients with chemotherapy induced DCM, and 1 (1%) patient with tachycardia induced DCM. Forty-seven (43%) DCM patients, and 81 (75%) NCCM patients underwent diagnostic cardiac magnetic resonance imaging (CMR). Seventeen patients (16%) in the DCM group and 15 patients (14%) in the NCCM group had congenital heart disease (CHD). The congenital heart diseases were predominantly minor septal defects chronic volume or pressure overload or operated at a young age.

The Left ventricular ejection fraction (LVEF) was primarily measured with the wall motion score index (89%) given the challenges in the delineation of the endomyocardial walls due to hypertrabeculation. DCM patients were more likely to have heart failure at primary presentation (40% vs. 28%; p = 0.049) and have lower systolic blood pressures (113 ± 19 vs. 127 ± 19; p < 0.001), which is due to the differences in medication use between the groups. DCM patient were more likely to have a sustained ventricular tachycardia (30% vs. 10%; p < 0.001).

NCCM patients were more likely to have a positive familial cardiomyopathy screening (55% vs. 24%; p < 0.001)and more likely to have known genetic variants (55% vs. 34%, p = 0.006), but the groups did not differ in prevalence of likely pathogenic or pathogenic (LP/P) variants. In the DCM group TTN variant was the most prevalent with 8 patients (36%), DSP variant in 4 patients (18%), and LMNA variant in 3 patients (14%). In the NCCM group MYH7 variant was seen in 12 (30%) and TTN in 8 (20%). Other gene variants only had a incidence of 1 or 2 patients.

**Table 1:** Baseline Characteristics of the Study Populations

Variable	DCM (n = 109)	NCCM (n = 109)	p-value
**Demographics**			
Age, years	45 [31–55]	45 [31–56]	0.88
Age at presentation, years	36 ± 14	38 ± 15	0.24
Male	58 (53)	58 (53)	1.00
**Ethnicity**			
Caucasian	92 (84)	94 (87)	0.58
African	3 (1)	4 (4)	0.72
Asian	4 (4)	2 (2)	0.68
**Comorbidities**			
Hypertension	24 (22)	21 (19)	0.59
Diabetes	21 (19)	2 (2)	< 0.001
Hypercholesterolemia	12 (11)	10 (9)	0.64
Coronary artery disease	15 (14)	11 (10)	0.39
Congenital heart disease	17 (16)	15 (14)	0.70
Stroke	1 (1)	3 (1)	0.62
**Primary presentation**			
Heart failure	43 (40)	30 (28)	0.049
Atrial arrhythmias	44 (41)	30 (28)	0.18
Sustained ventricular tachycardia	32 (30)	11 (10)	< 0.001
Ventricular fibrillation	5 (5)	11 (10)	0.18
Resuscitated	8 (7)	16 (15)	0.09
Stroke	3 (1)	3 (1)	1.00
Other	34 (32)	20 (18)	0.02
**Genetics**			
Positive familial screening CMP	19 (24)	48 (55)	< 0.001
Any variant	31 (34)	53 (55)	0.006
LP/P genetic variants	22 (24)	40 (42)	0.11
Sarcomere genes	18 (20)	38 (39)	0.004
Arrhythmia genes	1 (1)	4 (4)	0.37
Non-sarcomere, non-arrhythmia genes	9 (10)	10 (10)	0.94
Cardiac development genes	0 (0)	4 (4)	0.12
**Physical Examination**			
Height, m	1.75 ± 11	1.76 ± 10	0.72
Weight, kg	79 [67–97]	76 [67–89]	0.30
BMI, kg/m2	26 [23–30]	25 [23–28]	0.10
BSA, m2	1.92 ± 0.25	1.89 ± 0.22	0.34
Systolic BP, mmHg	113 ± 19	127 ± 19	< 0.001
Diastolic BP, mmHg	70 [60–80]	79 [70–81]	< 0.001
**Electrocardiography**			
Frequency, bpm	68 [60–78]	63 [56–70]	0.01
PQ, ms	169 [152–192]	165 [143–191]	0.54
QRS, ms	115 [101–149]	104 [92–124]	< 0.001
BBB	41 (38)	22 (20)	0.001
LVH	15 (15)	10 (9)	0.20
**Echocardiography**			
LA diameter, mm	40 [34–48]	39 [35–44]	0.49
LVED diameter, mm	60 [53–69]	58 [52–64]	0.09
LVES diameter, mm	51 ± 15	45 ± 13	0.02
LVEF, %	34 ± 11	41 ± 12	0.001
Aortic regurgitations	11 (11)	10 (10)	0.90
**Medication**			
Beta-receptor antagonist	100 (92)	80 (73)	< 0.001
ACE-inhibitor/ARB	94 (86)	72 (66)	< 0.001
Diuretics	75 (69)	35 (29)	< 0.001
Aldosteron receptor antagonist	67 (62)	26 (24)	< 0.001

* Continuous variables are summarised by mean ± SD or median (IQR), categorical variables are described as: n (%). ACE = angiotensin converting enzyme; AI = aortic valve insufficiency; Ao Asc = aorta ascendens; ARB = angiotensin receptor blocker; BBB = bundle branch block; BMI = body mass index; BP = blood pressure; BSA = body surface area; CMP = cardiomyopathy; LA = left atrium; LP/P: likely pathogenic or pathogenic; LVED = left ventricular end diastole; LVEF = left ventricular ejection fraction; LVES = left ventricular end systole; LVH = left ventricular hypertrophy SoV-Ao = sinuses of valsalva; STJ = sinotubular junction

### Measured aortic dimensions and prevalence of aortic dilatations

Measured aortic diameters and prevalence of aortic dilatations are presented in Table 2 A. An ascending aortic dilatation was observed in 8 (7%) patients. NCCM patients were not more likely to have a dilatation at the 3 measured aortic locations compared with the DCM patients; SoV-Ao (5% vs. 2%, p = 0.45), STJ (2% vs. 1%, p = 1.00), ascending aortic (7% vs. 5%, p = 0.46) or to have a dilatation in any of the three locations (10% vs. 5%, p = 0.15). Absolute diameter measurements did not significantly differ between DCM and NCCM.

**Table 2A:** Aortic dimensions and prevalence of dilatations in DCM and NCCM

Variable	DCM (n = 109)	NCCM (n = 109)	p-value
**Absolute aortic dimensions**			
SoV-Ao diameter, mm	30 ± 3	30 ± 5	0.59
STJ diameter, mm	29 ± 4	28 ± 4	0.15
Ao Asc diameter, mm	30 ± 4	30 ± 5	0.69
Bicuspid valve	1 (1)	0 (0)	1.00
Poor quality of echo	6 (6)	1 (1)	0.06
**Dilatations**			
Any dilation	5 (5)	11 (10)	0.15
SoV-Ao	2 (2)	5 (5)	0.45
STJ	1 (1)	2 (2)	1.00
Asc Ao	5 (5)	8 (7)	0.46

* Continuous variables are presented as mean ± SD, categorical variables are described as: n (%). SoV-Ao = sinuses of valsalva; STJ = sinotubular junction; Asc Ao = ascending aorta; Poor quality of echo = quality score < 3

### BSA-adjusted aortic dimensions

Table 2B shows the BSA-adjusted aortic dimensions for all patients and specifically stratified by sex in DCM and NCCM groups. Overall, the BSA adjusted aortic dimensions did not differ between DCM and NCCM patients. The adjusted dimension of DCM and NCCM patients differed among male participants at the level of the ascending aortic, mean of 14.7 [13.5–16.5] mm/m^2^ in DCM and 15.3 [14.1–17.1] mm/m^2^ in NCCM (p = 0.08). No significant differences in adjusted aortic dimensions at any level were noted between female DCM and NCCM patients. Figure 3 shows the overall and sex specific BSA adjusted aortic dimensions.

**Table 2B:** BSA Adjusted Aortic dimensions in DCM and NCCM

Variable	DCM (n = 109)	NCCM (n = 109)	p-value
**SoV-Ao mean**			
All cases	16.0 ± 2.2	16.0 ± 2.5	0.98
Male	15.5 [14.6–17.5]	15.7 [14.6–17.5]	0.11
Female	16.6 ± 2.3	15.7 ± 2.9	0.09
**STJ mean**			
All cases	15.1 [13.4–16.6]	14.6 [13.2–15.9]	0.26
Male	14.7 [13.0-16.5]	14.2 [13.5–15.8]	0.95
Female	15.4 ± 2.3	14.8 ± 2.6	0.18
**Ao Asc mean**			
All cases	15.4 [1.9-16.7]	15.3 [13.9–17.3]	0.73
Male	14.7 [13.5–16.5]	15.3 [14.1–17.1]	0.08
Female	16.0 [14.4–17.6]	15.0 [13.6–17.5]	0.23

* Continuous variables are summarised by mean ± SD or median (IQR). . SoV-Ao = sinuses of valsalva; STJ = sinotubular junction; Ao Asc = aorta ascendens

**Fig. 3a:**
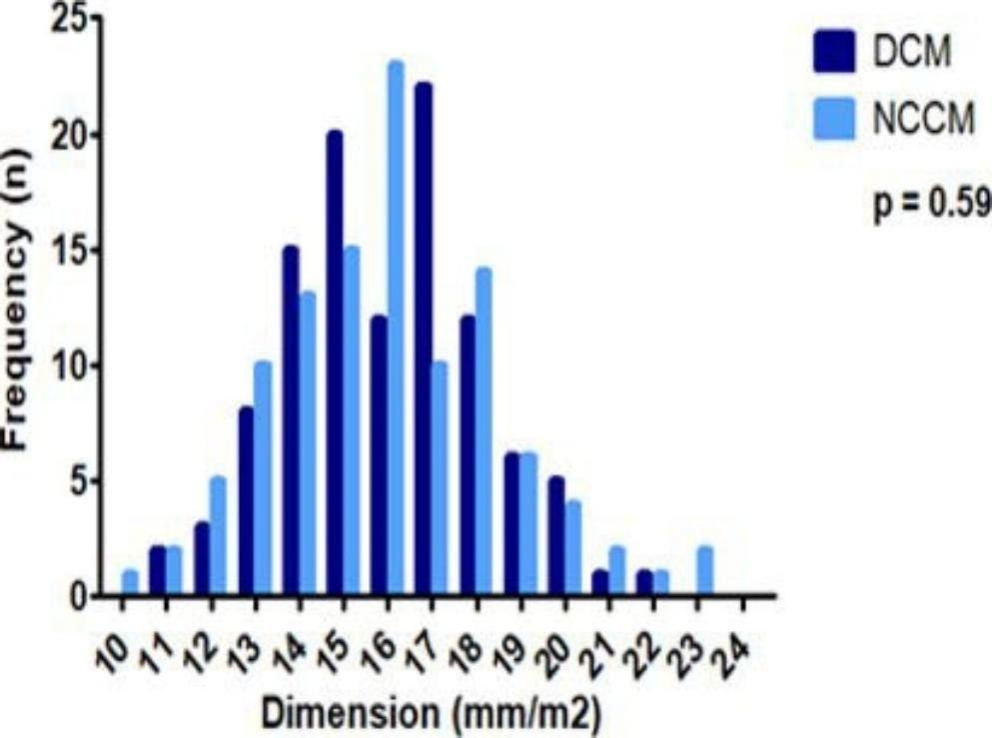
Adjusted Sinus of Valsalva Dimensions All Cases

**Fig. 3b:**
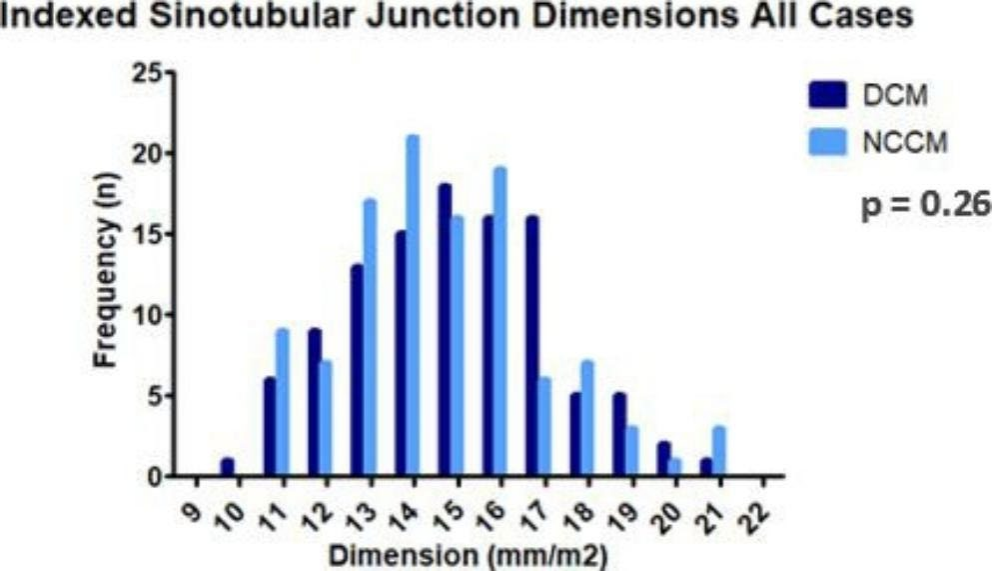
Adjusted Sinotubular Junction Dimensions All Cases

**Fig. 3c:**
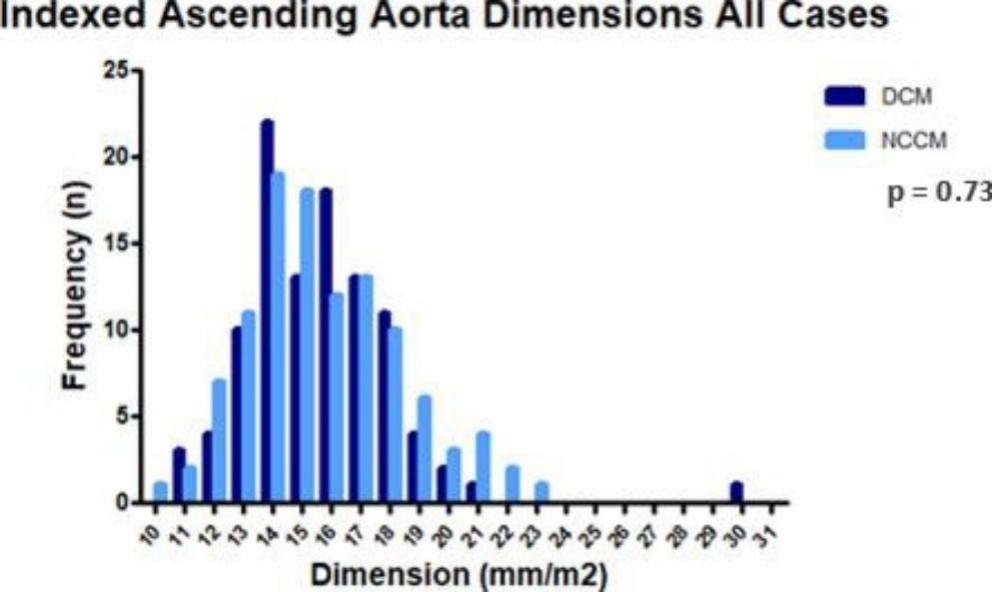
Adjusted Ascending Aorta Dimensions All Cases

### Comparing non-dilated to dilated ascending aorta groups in NCCM patients

Table 3 reports characteristics of NCCM patients divided in non-dilated-and-dilated aorta groups. Neither the dilated aorta group nor the non-dilated aorta group had a HCN4 variant. One patient had a MIB1 variant, but no bicuspid valve was found.

**Table 3:** Clinical Characteristics in Non-Dilated vs. Dilated Aorta NCCM patients

Variable	Non-Dilated aorta (n = 97)	Dilated aorta (n = 11)	p-value
**Demographics**			
Age, years	43 [30–55]	54 [41–61]	0.20
Age at presentation, years	37 ± 16	43 ± 12	0.23
Male	52 (54)	6 (55)	0.95
**Ethnicity**			
Caucasian	85 (89)	8 (73)	0.16
African	2 (2)	2 (18)	0.052
Asian	2 (2)	0 (0)	1.00
**Comorbidities**			
Hypertension	19 (20)	2 (18)	1.00
Diabetes	2 (2)	0 (0)	1.00
Hypercholesterolaemia	10 (10)	0 (0)	0.59
Coronary artery disease	10 (10)	1 (9)	1.00
Stroke	3 (3)	0 (0)	1.00
**Primary presentation**			
Heart failure	27 (28)	3 (27)	1.00
Arrhythmias	28 (29)	4 (36)	0.73
Stroke	3 (3)	0 (0)	1.00
Other	18 (19)	1 (9)	0.67
**Genetics**			
Positive familial screening CMP	43 (55)	5 (50)	1.00
Any variant	51 (58)	2 (22)	0.07
LP/P variants	38 (43)	2 (22)	0.25
Sarcomere genes	37 (42)	1 (9)	0.08
Arrhythmia genes	4 (5)	0 (0)	1.00
Non-sarcomere, non-arrhythmia genes	9 (10)	1 (11)	1.00
Cardiac development genes	4 (5)	0 (0)	1.00
**Physical Examination**			
Height, m	1.75 ± 10	1.76 ± 12	0.81
Weight, kg	79 ± 17	75 ± 16	0.38
BMI, kg/m2	26 ± 4	24 ± 4	0.25
BSA, m2	1.89 ± 0.22	1.85 ± 0.23	0.55
Systolic BP, mmHg	127 ± 19	124 ± 20	0.64
Diastolic BP, mmHg	79 [70–81]	80 [62–82]	0.93
**Electrocardiography**			
Frequency, bpm	62 [56–70]	65 [54–78]	0.54
**Echocardiography**			
LVEF, %	41 ± 12	35 ± 17	0.21
AI	8 (9)	2 (22)	0.22
SoV-Ao diameter, mm	29 ± 4	36 ± 5	< 0.001
STJ diameter, mm	27 ± 4	33 ± 4	< 0.001
Ao Asc diameter, mm	29 ± 4	37 ± 4	< 0.001
Bicuspid valve	0 (0)	0 (0)	
**Medication**			
Beta-receptor antagonist	74 (76)	6 (55)	0.15
ACE-inhibitor	65 (67)	7 (30)	1.00
Diuretics	29 (30)	3 (27)	1.00
Aldosterone	22 (23)	4 (36)	0.46

* Continuous variables are summarised by mean ± SD or median (IQR), categorical variables are described as: n (%). AI = aortic valve insufficiency; Ao Asc = aorta ascendens; BP = blood pressure; BMI = body mass index; BSA = body surface area; LP/P = likely pathogenic or pathogenic; LVEF = left ventricular ejection fraction; SoV-Ao = sinuses of valsalva; STJ = sinotubular junction

### Regression analyses

Table 4 shows the associations of different characteristics for the BSA-adjusted diameters of SoV-Ao, STJ and ascending aorta, adjusted for sex and age. NCCM was not significantly associated with larger aortic diameters (SoV-Ao; β = -0.0 mm, p = 0.99. STJ; β = -0.2 mm, p = 0.44. Asc Ao; β = 0.2 mm, p = 0.70). Black ethnicity was associated with a larger diameter in both STJ (β = 2.0 mm, p = 0.02) and in the ascending aorta (β = 2.7 mm, p = 0.002). The other characteristics were not significantly associated. Additionally, a poor quality of echo score was also not significantly associated (β = 0.48 mm, p = 0.84).

**Table 4:** Multivariate Linear Regression Analyses for Predictors of Aortic Diameters

	Adjusted Sinuses of Valsalva			Adjusted Sinotubular Junction			Adjusted Ascending Aorta			
Variables: all individually analysed with Sex and Age	β	CI 95%	p-value	β	CI 95%	p-value	β	CI 95%	p-value	
Noncompaction CMP	-0.002	-0.58–0.58	0.99	-0.23	-0.81–0.36	0.44	0.128	-0.52–0.77	0.70	
Caucasian (ref.: non-Caucasian)	-0.435	-1.28–0.41	0.31	-0.254	-1.10–0.59	0.56	-0.813	− 9.54 − 0.15	0.09	
African (ref.:non-African)	0.734	-1.04–2.61	0.42	2,038	0.38–3.70	0.02	2,715	0.99–4.44	0.002	
Asian (ref.:non-Asian)	0.73	-1.04–2.51	0.42	0.153	-0.76–1.06	0.87	0.331	-1.63–2.29	0.74	
Hypertension	-0.447	-1.19–0.29	0.24	-0.182	-0.92–0.55	0.63	-0.352	-1.15–0.45	0.39	
Familial CMP	0.129	-0.46–0.72	0.67	0.042	-0.57–0.65	0.89	-0.101	-0.77–0.57	0.77	
Any gene variant	-0.350	-0.96–0.26	0.26	-0.597	-1.24–0.04	0.07	-0.698	-1.41–0.01	0.06	
Sarcomere genes	-0.228	-0.90–0.44	0.50	-0.478	-1.17–0.21	0.17	-0.465	-1.27–0.34	0.25	
Arrhythmia genes	0.182	-1.10–1.46	0.78	-0.010	-1.20–1.18	0.99	0.505	-0.81–1.82	0.45	
Non-sarcomere, non-arrhythmia genes	0.303	-0.72–1.33	0.55	0.349	-0.56–1.26	0.45	0.327	-0.82–1.48	0.57	
Cardiac development gene variant	0.113	-1.57–1.80	0.89	0.508	-0.99–2.01	0.50	0.474	-0.11–2.05	0.55	
Systolic BP, mmHg	-0.008	-0.02–0.01	0.28	-0.001	-0.02–0.01	0.89	0.001	-0.02–0.02	0.86	
Bicuspid valve	-3.372	-7.49–0.74	0.12	-3.125	-7.39–1.14	0.15	-3.034	-7.70–1.63	0.20	

BP = blood pressure; CMP = cardiomyopathy; ref.: reference group.

## Discussion

This study found in 8 (7%) patients with an Asc Ao dilatation in patients with a NCCM: 5 (5.0%) patients with a SoV-Ao dilatation, 2 (2%) patients with a STJ dilatation, which were mainly mild. There was no significant difference in aortic dimensions between NCCM and age-sex matched DCM patients; not in absolute millimeters, nor after correcting for BSA, sex and age. Moreover, multiple linear regression found no significant association between NCCM and ascending aorta dilations. Remarkably, comparing the non-dilated ascending aorta group, no gene variants were found in the dilated group (58% vs. 0%, p = 0.006). Furthermore, black ethnicity showed a significant association with a larger adjusted ascending aorta (β = 2.7 mm, p = 0.002).

Studies on the prevalence of aortic dilation in hypertrophic and dilated cardiomyopathies that have been reported in the literature depend on the criteria used for defining ascending aortic dilatation. Their results range from 4.6% after adjusting for BSA, sex and age to 18% adjusting for only BSA [5–7]. However, the prevalence of aortic dilatation has not been previously investigated in NCCM. In our study, we found aortic dilatations in 7%, which is close to the 4.6% described in HCM and does not differ from DCM [5]. Thus, the prevalence of ascending aortic dilatations in NCCM is not atypical for a cardiomyopathy. The results of this study are also comparable to aortic dilatation prevalence in other, non-cardiomyopathy, populations. Babu et al. studied this in all patients who received TTE in their hospital during follow-up, finding a prevalence of 6.9%, almost the same as we observed in NCCM [18].

Gene variants associated with aortic dilatation are well known in Marfan syndrome and bicuspid aortic valve, and such variants can also occur in NCCM [3, 19, 20]. A study found an association between bicuspid aortic valve in noncompaction and MIB1 variant [21]. We did not find bicuspid aortic valves in this cohort so the prevalence remains unclear. We found that NCCM patients with an ascending aortic dilatation were less likely to have a genetic variant. Patients without a gene variant tend to get diagnosed at an older age, so age related risk factors for aortic dilatation such as hypertension and other comorbidities could be more present in the sporadic patient group. Furthermore, we found that black ethnicity is associated with a larger ascending aortic diameter. This suggests maybe that these patients have a more secondary forms of NCCM, i.e. hypertrabeculation (NCCM “lookalikes”?) as the prominent or excess trabeculations could indeed erroneously have been mistaken for noncompaction cardiomyopathy in blacks, athletes and patients with longstanding hypertension [22]. Another study found that the HCN4 gene variant, which causes a phenotype of NCCM, bradycardia and mitral valve disease, is also associated with a larger ascending aorta [9]. Although we could not conclude a difference in ascending aortic diameter between patients with NCCM and DCM, we cannot rule out a potential causal effect of the HCN4 variant on ascending aortic dilation as none of the included patients had HCN4 variant.

### Limitations

There are some limitations in this retrospective study. Firstly, the assessment of the aortic diameters was only done by echocardiographic images, because CT and MRI were available only in a subset of the patients. Aortic diameters may be over-or under-estimated when the quality of echo images was poor. However, we found no association between change in ascending aortic diameter and a poor quality of echo score. Incorrect alignment of the echocardiography could also have led to imprecise aortic diameters. Lastly, the study population was relatively small. Only 13 patients had an ascending aortic diameter meeting the diagnostic criteria for dilatation. Thus, although we did not find larger ascending aortic diameters amongst NCCM patients, we cannot rule out that a specific subgroup of NCCM patients, for example those presenting with a HCN4 variant, might be prone to ascending aortic dilation. Hence, it may underestimate the real prevalence of the ascending aortic dilatation in patients with a NCCM.

## Conclusions

In this cross-sectional cohort study the prevalence of an ascending aortic dilatation prevalence was 7% in NCCM patients, which were mild and not different from age-gender matched DCM controles. Therefore, routine aortic diameter screening therefore does not seem warranted in patients with NCCM.
